# Bis(4-methyl­morpholin-4-ium) tetra­bromidozincate(II)

**DOI:** 10.1107/S1600536811026766

**Published:** 2011-07-09

**Authors:** Cong-hu Peng, Yun-peng Li

**Affiliations:** a Department of Chemical & Environmental Engineering, Anyang Institute of Technology, Anyang 455000, People’s Republic of China; b Anyang Administration of Work Safety, Anyang 455000, People’s Republic of China

## Abstract

The title compound, (C_5_H_12_NO)_2_[ZnBr_4_], was synthesized by hydro­thermal reaction of ZnBr_2_ with 4-methyl­morpholine in a HBr/distilled water solution. Each of the two independent cations exhibits a chair conformation; the anion deviates slightly from an tetrahedral configuration. The Zn—Br distances in the anion are in the range of 2.3996 (9)–2.4247 (9) Å. All of the amine H atoms are involved in bifurcated inter­molecular N—H⋯Br hydrogen bonds, building up a trimer.

## Related literature

For the preparation of amino coordination compounds, see: Fu *et al.* (2009 ▶); Aminabhavi *et al.* (1986 ▶). 
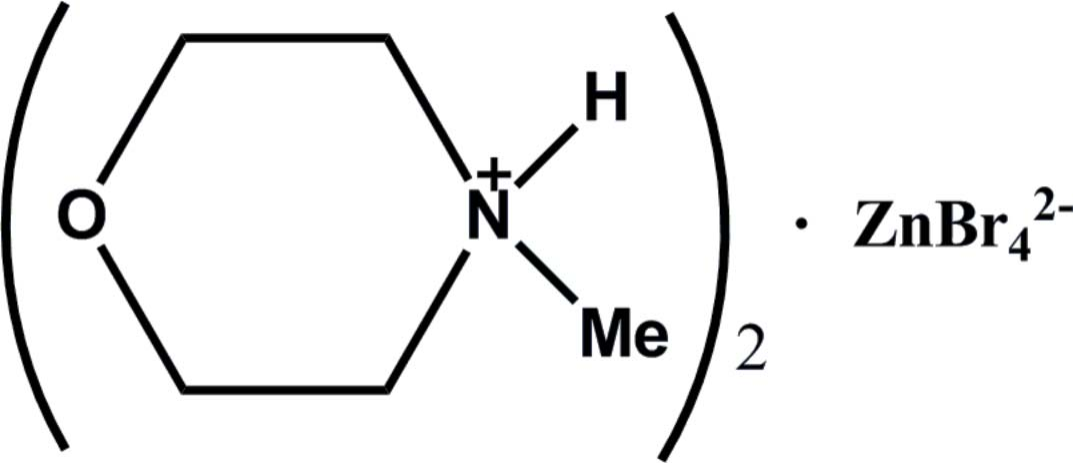


## Experimental

### 

#### Crystal data

(C_5_H_12_NO)_2_[ZnBr_4_]
*M* *_r_* = 589.32
Monoclinic, 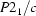

*a* = 7.5000 (15) Å
*b* = 20.925 (4) Å
*c* = 12.670 (3) Å
β = 103.33 (3)°
*V* = 1934.8 (7) Å^3^
*Z* = 4
Mo *K*α radiation
μ = 9.53 mm^−1^
*T* = 298 K
0.30 × 0.02 × 0.01 mm

#### Data collection

Rigaku Mercury2 diffractometer
Absorption correction: multi-scan (*CrystalClear*; Rigaku, 2005 ▶) *T*_min_ = 0.810, *T*_max_ = 0.900
19809 measured reflections
4439 independent reflections
3118 reflections with *I* > 2σ(*I*)
*R*_int_ = 0.079

#### Refinement

*R*[*F* ^2^ > 2σ(*F* ^2^)] = 0.047
*wR*(*F* ^2^) = 0.096
*S* = 1.07
4439 reflections
174 parameters
H-atom parameters constrained
Δρ_max_ = 0.58 e Å^−3^
Δρ_min_ = −0.78 e Å^−3^

### 

Data collection: *CrystalClear* (Rigaku, 2005 ▶); cell refinement: *CrystalClear*; data reduction: *CrystalClear*; program(s) used to solve structure: *SHELXS97* (Sheldrick, 2008 ▶); program(s) used to refine structure: *SHELXL97* (Sheldrick, 2008 ▶); molecular graphics: *SHELXTL* (Sheldrick, 2008 ▶); software used to prepare material for publication: *SHELXTL*.

## Supplementary Material

Crystal structure: contains datablock(s) I, global. DOI: 10.1107/S1600536811026766/ru2007sup1.cif

Structure factors: contains datablock(s) I. DOI: 10.1107/S1600536811026766/ru2007Isup2.hkl

Additional supplementary materials:  crystallographic information; 3D view; checkCIF report

## Figures and Tables

**Table 1:** Hydrogen-bond geometry (Å, °)

*D*—H⋯*A*	*D*—H	H⋯*A*	*D*⋯*A*	*D*—H⋯*A*
N1—H1⋯Br2^i^	0.90	2.70	3.427 (4)	138
N1—H1⋯Br3^i^	0.90	2.87	3.541 (4)	132
N2—H2⋯Br4	0.90	2.72	3.504 (4)	147
N2—H2⋯Br1	0.90	3.08	3.714 (4)	129

Symmetry code: (i) 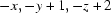.

**Fig. 1.**
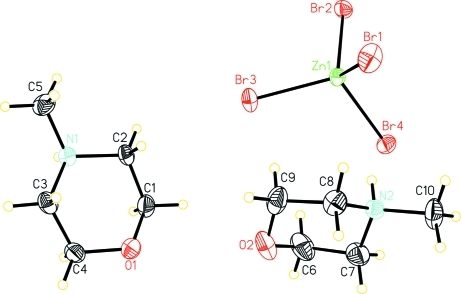
Molecular view of the title compound with the atomic numbering scheme. Displacement ellipsoids are drawn at the 30% probability level.

**Fig. 2.**
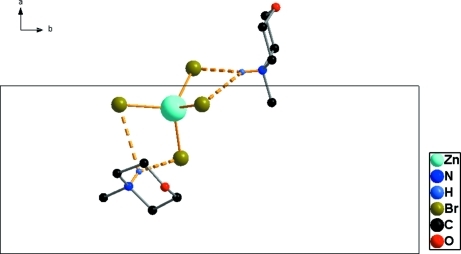
The crystal packing of the title compound viewed along the c axis showing the one-dimensionnal hydrogen bondings chain (dashed line). Hydrogen atoms not involved in hydrogen bonding have been omitted for clarity.

## supplementary crystallographic information

### Comment

The amino derivatives have found wide range of applications in material science, such as magnetic, fluorescent and dielectric behaviors. And there has been an increased interest in the preparation of amino coordination compound (Aminabhavi *et al.,* 1986; Fu, *et al.* 2009). We report here the crystal structure of the title compound, *Bis-*(4-methylmorpholin-4-ium) tetrabromidozincate(II).

The asymmetric unit is composed of one ZnBr_4_^2-^ anion and two 4-methylmorpholin-4-ium cations (Fig.1). Both the amine N atoms were protonated, thus indicating two positive charge in all. And the ZnBr_4_^2-^ anion was showing two negative charge to make the charge balance. The geometric parameters of the title compound are in the normal range.

In the crystal structure, all the H atoms of amine groups are involved in intermolecular N—H···Br hydrogen bonds building up a trimer (Table 1 and Fig.2).

### Experimental

A mixture of 4-methylmorpholine (0.4 mmol), ZnBr_2_ (0.4 mmol) and HBr/distilled water (10ml,1:3) sealed in a Teflon-lined stainless steel vessel, was maintained at 100 °C. Colorless needle crystals suitable for X-ray analysis were obtained after 3 days.

### Refinement

All H atoms attached to C atoms were fixed geometrically and treated as riding with C-H = 0.97 Å(methylene) and C-H = 0.96 Å(methyl) with *U*_iso_(H) = 1.2*U*_eq_(methylene) and *U*_iso_(H) = 1.5*U*_eq_(methyl). The positional parameters of the H atoms (N1, N2) were refined freely. And in the last stage of the refinement, it were restrained with the H—N = 0.90 (2)Å), with *U_iso_*(H)=1.2*U_eq_*(N).

### Crystal data

**Table:**

(C_5_H_12_NO)_2_[ZnBr_4_]	*F*(000) = 1136
*M**_r_* = 589.32	*D*_x_ = 2.023 Mg m^−^^3^
Monoclinic, *P*2_1_/*c*	Mo *K*α radiation, λ = 0.71073 Å
Hall symbol: -P 2ybc	Cell parameters from 4439 reflections
*a* = 7.5000 (15) Å	θ = 3.1–27.5°
*b* = 20.925 (4) Å	µ = 9.53 mm^−^^1^
*c* = 12.670 (3) Å	*T* = 298 K
β = 103.33 (3)°	Needle, colorless
*V* = 1934.8 (7) Å^3^	0.30 × 0.02 × 0.01 mm
*Z* = 4	

### Data collection

**Table:**

Rigaku Mercury2 diffractometer	4439 independent reflections
Radiation source: fine-focus sealed tube	3118 reflections with *I* > 2σ(*I*)
graphite	*R*_int_ = 0.079
Detector resolution: 13.6612 pixels mm^-1^	θ_max_ = 27.5°, θ_min_ = 3.1°
CCD profile fitting scans	*h* = −9→9
Absorption correction: multi-scan (*CrystalClear*; Rigaku, 2005)	*k* = −27→27
*T*_min_ = 0.810, *T*_max_ = 0.900	*l* = −16→16
19809 measured reflections	

### Refinement

**Table:**

Refinement on *F*^2^	Primary atom site location: structure-invariant direct methods
Least-squares matrix: full	Secondary atom site location: difference Fourier map
*R*[*F*^2^ > 2σ(*F*^2^)] = 0.047	Hydrogen site location: inferred from neighbouring sites
*wR*(*F*^2^) = 0.096	H-atom parameters constrained
*S* = 1.07	*w* = 1/[σ^2^(*F*_o_^2^) + (0.024*P*)^2^ + 1.999*P*] where *P* = (*F*_o_^2^ + 2*F*_c_^2^)/3
4439 reflections	(Δ/σ)_max_ = 0.001
174 parameters	Δρ_max_ = 0.58 e Å^−^^3^
0 restraints	Δρ_min_ = −0.78 e Å^−^^3^

### Special details

**Table:**

Geometry. All esds (except the esd in the dihedral angle between two l.s. planes) are estimated using the full covariance matrix. The cell esds are taken into account individually in the estimation of esds in distances, angles and torsion angles; correlations between esds in cell parameters are only used when they are defined by crystal symmetry. An approximate (isotropic) treatment of cell esds is used for estimating esds involving l.s. planes.
Refinement. Refinement of F^2^ against ALL reflections. The weighted R-factor wR and goodness of fit S are based on F^2^, conventional R-factors R are based on F, with F set to zero for negative F^2^. The threshold expression of F^2^ > 2sigma(F^2^) is used only for calculating R-factors(gt) etc. and is not relevant to the choice of reflections for refinement. R-factors based on F^2^ are statistically about twice as large as those based on F, and R- factors based on ALL data will be even larger.

### Fractional atomic coordinates and isotropic or equivalent isotropic displacement parameters (Å^2^)

**Table:**

	*x*	*y*	*z*	*U*_iso_*/*U*_eq_	
Zn1	0.13367 (8)	0.63934 (3)	0.72630 (5)	0.03501 (16)	
N1	0.0938 (5)	0.54509 (19)	1.2863 (3)	0.0349 (10)	
H1	0.0825	0.5023	1.2819	0.042*	
Br2	−0.10482 (8)	0.59847 (3)	0.58209 (4)	0.04782 (17)	
Br3	0.11003 (9)	0.58049 (3)	0.88737 (4)	0.04937 (18)	
C3	0.2234 (8)	0.5701 (3)	1.3845 (4)	0.0463 (14)	
H3A	0.1917	0.5532	1.4491	0.056*	
H3B	0.2144	0.6163	1.3864	0.056*	
Br4	0.43069 (7)	0.62464 (3)	0.68930 (5)	0.04825 (17)	
Br1	0.11182 (10)	0.75261 (3)	0.75420 (6)	0.0650 (2)	
O1	0.4678 (5)	0.57277 (19)	1.2887 (3)	0.0552 (11)	
C5	−0.0990 (8)	0.5644 (3)	1.2811 (5)	0.0537 (16)	
H5A	−0.1349	0.5495	1.3448	0.081*	
H5B	−0.1778	0.5459	1.2179	0.081*	
H5C	−0.1086	0.6101	1.2772	0.081*	
C4	0.4175 (8)	0.5510 (3)	1.3833 (4)	0.0507 (15)	
H4A	0.5011	0.5688	1.4465	0.061*	
H4B	0.4282	0.5049	1.3873	0.061*	
C2	0.1551 (7)	0.5645 (2)	1.1864 (4)	0.0379 (13)	
H2A	0.1432	0.6104	1.1768	0.045*	
H2B	0.0780	0.5441	1.1233	0.045*	
C1	0.3508 (8)	0.5452 (3)	1.1963 (5)	0.0505 (15)	
H1A	0.3604	0.4990	1.2013	0.061*	
H1B	0.3896	0.5585	1.1318	0.061*	
N2	0.5985 (6)	0.7329 (2)	0.8998 (3)	0.0390 (10)	
H2	0.5105	0.7118	0.8522	0.047*	
O2	0.6025 (7)	0.6575 (2)	1.0880 (3)	0.0724 (14)	
C8	0.5215 (9)	0.7569 (3)	0.9907 (5)	0.0528 (16)	
H8A	0.4179	0.7847	0.9626	0.063*	
H8B	0.6136	0.7814	1.0408	0.063*	
C9	0.4608 (9)	0.7010 (3)	1.0494 (5)	0.0628 (19)	
H9A	0.4146	0.7168	1.1099	0.075*	
H9B	0.3613	0.6790	1.0004	0.075*	
C7	0.7407 (9)	0.6834 (3)	0.9412 (5)	0.0641 (18)	
H7A	0.7824	0.6648	0.8810	0.077*	
H7B	0.8451	0.7031	0.9899	0.077*	
C10	0.6659 (10)	0.7853 (3)	0.8407 (5)	0.0680 (19)	
H10A	0.7112	0.7678	0.7819	0.102*	
H10B	0.7628	0.8077	0.8893	0.102*	
H10C	0.5673	0.8143	0.8126	0.102*	
C6	0.6643 (11)	0.6325 (3)	0.9995 (6)	0.076 (2)	
H6A	0.5630	0.6118	0.9498	0.091*	
H6B	0.7578	0.6006	1.0254	0.091*	

### Atomic displacement parameters (Å^2^)

**Table:**

	*U*^11^	*U*^22^	*U*^33^	*U*^12^	*U*^13^	*U*^23^
Zn1	0.0398 (3)	0.0309 (3)	0.0357 (3)	−0.0033 (3)	0.0116 (3)	−0.0006 (3)
N1	0.043 (2)	0.029 (2)	0.033 (2)	−0.0024 (19)	0.0091 (19)	−0.0058 (19)
Br2	0.0445 (3)	0.0533 (4)	0.0409 (3)	−0.0084 (3)	0.0002 (3)	0.0049 (3)
Br3	0.0710 (4)	0.0452 (3)	0.0344 (3)	−0.0144 (3)	0.0171 (3)	0.0006 (3)
C3	0.058 (4)	0.045 (3)	0.034 (3)	−0.006 (3)	0.007 (3)	−0.004 (3)
Br4	0.0418 (3)	0.0538 (4)	0.0530 (4)	0.0060 (3)	0.0189 (3)	0.0008 (3)
Br1	0.0816 (5)	0.0295 (3)	0.0932 (5)	−0.0003 (3)	0.0392 (4)	−0.0060 (3)
O1	0.047 (2)	0.064 (3)	0.054 (3)	−0.016 (2)	0.011 (2)	0.010 (2)
C5	0.046 (3)	0.062 (4)	0.057 (4)	0.011 (3)	0.019 (3)	−0.010 (3)
C4	0.050 (4)	0.055 (4)	0.042 (3)	−0.008 (3)	0.001 (3)	0.010 (3)
C2	0.051 (3)	0.032 (3)	0.031 (3)	−0.007 (2)	0.009 (2)	0.004 (2)
C1	0.058 (4)	0.052 (4)	0.045 (3)	−0.009 (3)	0.018 (3)	0.005 (3)
N2	0.043 (3)	0.039 (3)	0.033 (2)	−0.004 (2)	0.007 (2)	−0.002 (2)
O2	0.102 (4)	0.071 (3)	0.043 (3)	−0.001 (3)	0.015 (3)	0.015 (2)
C8	0.060 (4)	0.053 (4)	0.049 (4)	−0.001 (3)	0.021 (3)	−0.014 (3)
C9	0.073 (5)	0.074 (5)	0.049 (4)	−0.025 (4)	0.029 (3)	−0.015 (4)
C7	0.058 (4)	0.075 (5)	0.062 (4)	0.024 (4)	0.018 (3)	0.012 (4)
C10	0.092 (5)	0.058 (4)	0.060 (4)	−0.023 (4)	0.029 (4)	0.010 (3)
C6	0.099 (6)	0.062 (5)	0.066 (5)	0.027 (4)	0.019 (4)	0.019 (4)

### Geometric parameters (Å, °)

**Table:**

Zn1—Br4	2.3996 (9)	C1—H1A	0.9700
Zn1—Br2	2.4005 (11)	C1—H1B	0.9700
Zn1—Br1	2.4075 (9)	N2—C10	1.480 (7)
Zn1—Br3	2.4247 (9)	N2—C8	1.490 (6)
N1—C3	1.487 (6)	N2—C7	1.493 (7)
N1—C5	1.488 (7)	N2—H2	0.9002
N1—C2	1.499 (6)	O2—C9	1.398 (8)
N1—H1	0.9000	O2—C6	1.409 (8)
C3—C4	1.513 (8)	C8—C9	1.511 (8)
C3—H3A	0.9700	C8—H8A	0.9700
C3—H3B	0.9700	C8—H8B	0.9700
O1—C4	1.412 (6)	C9—H9A	0.9700
O1—C1	1.414 (7)	C9—H9B	0.9700
C5—H5A	0.9600	C7—C6	1.484 (9)
C5—H5B	0.9600	C7—H7A	0.9700
C5—H5C	0.9600	C7—H7B	0.9700
C4—H4A	0.9700	C10—H10A	0.9600
C4—H4B	0.9700	C10—H10B	0.9600
C2—C1	1.499 (7)	C10—H10C	0.9600
C2—H2A	0.9700	C6—H6A	0.9700
C2—H2B	0.9700	C6—H6B	0.9700
			
Br4—Zn1—Br2	111.59 (4)	O1—C1—H1B	109.3
Br4—Zn1—Br1	104.63 (3)	C2—C1—H1B	109.3
Br2—Zn1—Br1	113.48 (3)	H1A—C1—H1B	108.0
Br4—Zn1—Br3	110.52 (4)	C10—N2—C8	112.3 (5)
Br2—Zn1—Br3	105.87 (3)	C10—N2—C7	113.1 (5)
Br1—Zn1—Br3	110.84 (3)	C8—N2—C7	109.6 (4)
C3—N1—C5	112.5 (4)	C10—N2—H2	107.9
C3—N1—C2	110.1 (4)	C8—N2—H2	109.1
C5—N1—C2	111.9 (4)	C7—N2—H2	104.5
C3—N1—H1	115.9	C9—O2—C6	109.0 (5)
C5—N1—H1	101.0	N2—C8—C9	109.5 (5)
C2—N1—H1	105.0	N2—C8—H8A	109.8
N1—C3—C4	110.1 (4)	C9—C8—H8A	109.8
N1—C3—H3A	109.6	N2—C8—H8B	109.8
C4—C3—H3A	109.6	C9—C8—H8B	109.8
N1—C3—H3B	109.6	H8A—C8—H8B	108.2
C4—C3—H3B	109.6	O2—C9—C8	112.6 (5)
H3A—C3—H3B	108.2	O2—C9—H9A	109.1
C4—O1—C1	109.5 (4)	C8—C9—H9A	109.1
N1—C5—H5A	109.5	O2—C9—H9B	109.1
N1—C5—H5B	109.5	C8—C9—H9B	109.1
H5A—C5—H5B	109.5	H9A—C9—H9B	107.8
N1—C5—H5C	109.5	C6—C7—N2	110.3 (5)
H5A—C5—H5C	109.5	C6—C7—H7A	109.6
H5B—C5—H5C	109.5	N2—C7—H7A	109.6
O1—C4—C3	111.7 (4)	C6—C7—H7B	109.6
O1—C4—H4A	109.3	N2—C7—H7B	109.6
C3—C4—H4A	109.3	H7A—C7—H7B	108.1
O1—C4—H4B	109.3	N2—C10—H10A	109.5
C3—C4—H4B	109.3	N2—C10—H10B	109.5
H4A—C4—H4B	107.9	H10A—C10—H10B	109.5
N1—C2—C1	109.9 (4)	N2—C10—H10C	109.5
N1—C2—H2A	109.7	H10A—C10—H10C	109.5
C1—C2—H2A	109.7	H10B—C10—H10C	109.5
N1—C2—H2B	109.7	O2—C6—C7	111.4 (6)
C1—C2—H2B	109.7	O2—C6—H6A	109.3
H2A—C2—H2B	108.2	C7—C6—H6A	109.3
O1—C1—C2	111.6 (5)	O2—C6—H6B	109.3
O1—C1—H1A	109.3	C7—C6—H6B	109.3
C2—C1—H1A	109.3	H6A—C6—H6B	108.0

### Hydrogen-bond geometry (Å, °)

**Table:**

*D*—H···*A*	*D*—H	H···*A*	*D*···*A*	*D*—H···*A*
N1—H1···Br2^i^	0.90	2.70	3.427 (4)	138
N1—H1···Br3^i^	0.90	2.87	3.541 (4)	132
N2—H2···Br4	0.90	2.72	3.504 (4)	147
N2—H2···Br1	0.90	3.08	3.714 (4)	129

Symmetry codes: (i) −*x*, −*y*+1, −*z*+2.
